# Consumer demand for novel fruit and vegetable products with extended shelf lives in East Africa: a multinational multi-product analysis

**DOI:** 10.1017/S136898002100478X

**Published:** 2022-06

**Authors:** Johanna Tepe, Marwan Benali, Dominic Lemken

**Affiliations:** 1 Department of Agricultural Economics and Rural Development, Marketing of Food and Agricultural Products, University of Göttingen, Platz der Göttinger Sieben 5, 37073 Göttingen, Germany; 2 World Vegetable Center, West and Central Africa – Coastal and Humid Regions, Cotonou, Benin

**Keywords:** Processed fruits and vegetables, Consumer demand, Sensory analysis, Willingness to pay, East Africa

## Abstract

**Objective:**

To evaluate the potential of products made out of underutilised fruits and vegetables for closing seasonal nutritional gaps among rural and urban consumers in East Africa.

**Design:**

The multinational analysis combines sensory testing and experimental auctions to assess consumers’ perceptions and willingness to pay (WTP) for 6 different fruit and vegetable products.

**Setting:**

Open markets in rural and urban areas in Kenya, Tanzania and Uganda.

**Participants:**

There were 939 male and female adults who were at least 18 years old.

**Results:**

Tobit models for each product show that besides sensory perception, similar socio-demographic characteristics influence consumers’ WTP for these products in all 3 countries. The products are especially liked among younger, male and urban consumers.

**Conclusion:**

We conclude that there is demand and a potential market for processed fruit and vegetable products based on indigenous raw material in East Africa. The products, thus, have promising potential to improve nutrition, especially during off-season conditions when access to fresh produce is limited.

Malnutrition is still widespread in East Africa. Up to one-fourth of the population is overweight, and one-fifth is at risk of dying from cardiovascular diseases. Concurrently, undernourishment in Kenya, Tanzania and Uganda varies at approximately 35 % with more than 28 % of women of reproductive age suffering from anaemia due to Fe deficiencies^(1)^. Fruit and vegetable intake, limited to only 2 to 4 times per week^(2)^, is low among the food-insecure population^(3)^. The findings of Keding *et al.* ^(4)^ indicated that seasonality further impairs the nutritional status of the population. In addition, high amounts of nutritious fruits and vegetables are lost due to inappropriate storage and processing techniques. The FAO^(5)^ reported losses of up to 50 % in sub-Saharan Africa. Compared with other foods, fruits and vegetables are especially impacted by losses due to their high perishability, an issue that is further exacerbated by the warm and humid climates in East Africa. A first pilot study found a link between food losses and nutrient deficiencies in children in Kenya^(5)^. This study concluded that food loss reductions can, respectively, satisfy up to 24 % and 33 % of the Fe and vitamin C requirements of children under 5^(5)^. In Kenya, fresh fruits and vegetables, such as cowpea leaves and guavas, which are rich in nutrients^(6,7)^, are lost due to poor postharvest handling techniques and processing. Major challenges include the short shelf life for fresh produce (e.g. a maximum of 5 d for guava) and inadequate preservation techniques^(6,8)^.

The demand for processed fruit and vegetable products can contribute to lowering postharvest losses and improving nutrition simultaneously. Processing allows producers to diversify their income and provides consumers access to nutritious food independent of the harvesting season. Van der Lans *et al*.^(9)^ emphasised the potential of processing to bridge seasonal nutritional gaps and lower postharvest losses. Furthermore, Okello *et al.* ^(10)^ highlighted the benefits of the solar drying of vegetables. It is an effective way to ensure year-round access to nutritious vegetables. Food processing can also help increase the likability of a fruit or vegetable, as evidence from Nigeria revealed astringent compounds in cashew apples as a major obstacle in acceptance^(11)^ and processing techniques being able to lower the astringency^(12)^.

Moreover, processed fruits and vegetables fall into the currently undergoing shift towards more processed foods in Africa and globally^(13)^. A review on dietary behaviour in Kenya found that while fruit and vegetable consumption was low, the consumption of sugar-sweetened beverages and processed/fried foods was widespread^(14)^. The current trend is predicted to cause considerable health burdens^(15)^. Processing fruits and vegetables sensibly could simultaneously appeal to consumers currently seeking ready-to-eat products and provide healthier alternatives for the sugar-sweetened beverages and snacks currently available in the market.

There are limited published data and evidence on the consumer evaluation of the sensory attributes of processed fruits and vegetables and their demand in East Africa. Okello *et al*.^(10)^ found that consumers’ demand for processed cowpea leaves exists, but a large share of consumers were unaware of the benefits this product provides; their study targeted urban and peri-urban consumers. It is likely that the demand will be different in rural, resource-poor households with less access to diverse markets. Fruit and vegetable products can only contribute to overcoming seasonal gaps in micronutrient supply if they are accepted as well as regularly demanded and consumed.

The objective of this study is to evaluate the potential of products made out of underutilised fruits and vegetables for the purpose of closing seasonal nutritional gaps among rural and urban consumers in East Africa. To respond to this challenge, food technologists and plant scientists have developed processed fruit and vegetable products that can bridge nutritional gaps and have extended shelf lives. The consumer acceptance of such products will be tested in this study. The test products include guava nectar, cowpea leaf soup mix, African nightshade relish, dried African nightshade, dried cashew apple and jackfruit juice. The raw fruits and vegetables necessary for the products were selected due to their high nutritional values and because they are currently being neglected. Consumers from both urban and rural areas will be targeted and the influence of the location will be assessed.

The remainder of the paper is organised as follows. Section 2 describes the methods applied in the study. The section includes a brief description of the products tested, the study site and the target populations as well as a more comprehensive description of the sensory evaluation, willingness to pay (WTP) experiment and the empirical model. Section 3 will present the results, which will be discussed in Section 4. Section 5 concludes the paper.

## Methods

### Products tested

To address the research objectives, 6 different fruit- and vegetable-based products were tested in Kenya, Tanzania and Uganda (Table 1).

**Table 1:** Products tested by country

	Kenya		Tanzania			Uganda
	Cowpea leaf soup mix	Guava nectar	Dried cashew apples	African nightshade relish	Dried African nightshade	Jackfruit juice
Off-season fresh produce	July to August October to March	July to February	January to September	June to December	June to December	none
Packaging	Kraft paper	Plastic bottle	Plastic bag	Plastic container	Plastic bag	Plastic bottle
Shelf life (months)	5	3–5	6	under analysis	6	6

In Kenya, this included guava nectar and cowpea leaf soup mix. The guava nectar was prepared from guava pulp, moringa leaf juice extract, sugar, citric acid and preservatives. Besides cowpea leaves, the soup contained a mixture of starch, coriander, tomato, onions, vegetable oil and garlic. Both products were processed and packaged at the Department of Food Science of the University of Nairobi. The soup was prepared in the morning and stored in ThermoFlasks. For preparation, 50 g of soup powder was mixed with 10 ml of cold water. The mixture was set on a stove and another 490 ml of water was added. While stirring, the mixture was boiled for 5 min.

In Tanzania, an African nightshade relish, dried African nightshade and sun-dried cashew apples were tested. All 3 products were processed and packaged at the Department of Food Biotechnology of the Nelson Mandela African Institution of Science and Technology in Arusha. The African nightshade relish was fermented and pepper, turmeric, garlic, cardamom, cooking oil, salt, onions and carrots were added. The dried African nightshade was freshly prepared each morning in a traditional way before the experiments started. The dried leaves were fried in a pan using sunflower oil. Onions, green pepper, tomatoes and yellow chili were added. The prepared dried African nightshade was then stored in hot pots and taken to the market. The cashew apples were blanched, sliced, immersed in 70 % sucrose and then sun-dried. The dried cashew apples were rich in carotenoids (0·28 g/100 g dry basis), vitamin C (0·73 g/100 g dry basis) and tannins (266·59 mg/100 g dry basis)^(16)^. The tannin content was significantly reduced compared with the fresh fruit. The moisture content of the product was measured at 13·81 %. The nutritive value decreased over time but was still at an acceptable level after 60 d^(16)^. The African nightshade and the dried cashew apples were stored in cool boxes.

In Uganda, jackfruit juice was tested. The juice was prepared using jackfruit pulp, preservatives and sugar. The products were processed and packaged at the Department of Food Technology and Nutrition at Makerere University Kampala. The juice bottles were stored in cool boxes during the day.

In all products, ingredients that can be harmful to human health when consumed excessively, such as sugar and salt were kept low. Pre-trials were conducted for the guava nectar to determine the lowest level of sugar that was still accepted by the consumers.

### Study site, set-up and participants

Data collection was conducted between October 2019 and February 2020 in Kenya, Tanzania and Uganda. In total, 1225 participants were questioned. On several occasions’, environmental influences, such as sudden downpours and strong winds, caused disruptions. To ensure validity, we dropped these days from the analysis. Some participants were dropped due to incomplete questionnaires. After data cleaning, 939 participants remained for further analysis.

Within all 3 countries, an urban and a rural area were selected to test the products. Both areas were chosen purposively, based on a justifiable effort to reach the target population. The major aim of the project was to bring nutritious products to rural areas, where micronutrient deficiencies are often more severe than in urban areas. However, when introducing novel products in the markets, it is often easier for producers to start distribution in urban areas, where the infrastructure is more advanced. In Kenya, Nairobi and the Taita-Taveta region were chosen to represent urban and rural consumers, respectively; in Tanzania, the Morogoro Municipal Council and the Morogoro District Council were selected, respectively; and in Uganda, Kampala and Kayunga were selected, respectively. Within each study region, respondents were targeted at open markets; within each region, 4–5 markets were selected due to their convenience aspects. Markets were defined as the usual place where many sellers met once or twice a week to sell their produce. Respondents were approached when leaving the market and independently of their gender, based on convenience aspects such as readiness to participate. Different markets were chosen to cover different market days (there was usually one market open per day) and to ensure that the respondents had not heard about the products already from family, colleagues or friends.

Respondents had to meet the qualification criteria to participate in the survey. These criteria included being at least 18 years old, being free of diabetes and food sensitivities, possessing the responsibility to make food-purchasing decisions in the household and being interested in testing the target products. All information was self-reported. However, the participants were informed that giving false information could be consequential to their health. Only respondents who met all the criteria were eligible. Qualified respondents were informed about their right to leave the survey at any time; they were also asked to give their written consent. Respondents agreeing to participate received a participation fee as a token of appreciation and to build their financial means to participate in the auction. The participation fee was set at double the expected WTP. For example, in Kenya, the expected WTP for the guava nectar and cowpea leaf soup mix was 30 Kenyan shillings (KSH). This led to a participation fee of 120 KSH (1·16 US$). In Tanzania participants received 2400 Tanzanian shillings (TSH) (1·05 US$) and in Uganda 4000 Ugandan shillings (UGX) (1·08 US$). The exchange rates at the time of the study were used. The procedure was based on that used by De Groote *et al.* ^(17)^. The enumerators started the survey with the sensory analysis.

### Sensory testing

For sensory testing, consumers were asked to rate the products on a 5-point Likert scale: 1 = dislike it very much, 2 = dislike it, 3 = neither like it nor dislike it, 4 = like it and 5 = like it very much. The 5-point Likert scale has already been applied in previous surveys in East Africa and was proven to be easier to understand by respondents with no or limited education^(18,19)^. A small sample of each product was served in a plastic cup. Between products, respondents were asked to rinse their mouth with water. The product order was randomised to avoid first-sample bias. The tested sensory characteristics included colour, aroma, texture in the mouth, taste and general appearance.

### Information treatment

After sensory testing and before the WTP analysis, every second participant received additional information about the products. Earlier studies on WTP for food products in East Africa found the provision of information to translate into higher WTP^(19–21)^; the studies used different methods to present the information. To analyse the effect of the information, half of the participants in each country were informed about the nutritional aspects and/or convenience and shelf life characteristics of the products. We do not believe there were systematic differences between the 2 groups, although we have noticed tendencies in the sensory perception of the cowpea leaf soup mix and jackfruit juice, yet towards different sides (see online Supplemental Table A.1). As each product had its own specific characteristics, the given information differed. Besides information about the nutritional value of the products, participants were informed about the year-around availability of the product compared with the seasonality of the raw fruits and vegetables. The only exception was jackfruit juice, as jackfruits can be harvested year-round. In addition to the nutritional value information and the shelf life benefits, information about the cowpea leaf soup mix, African nightshade relish and dried African nightshade included convenience aspects, such as being able to prepare those products much faster and easier than preparing the fresh vegetables. The information was presented to the respondents using images. As an example, the images for the cowpea leaf soup mix are presented in online Supplemental Appendix Fig. A.4. The enumerators were carefully trained on how to explain the information.

### Willingness to pay

A popular experimental method to assess consumers WTP is the Becker–DeGroot–Marschak (BDM) auction. BDM auctions offer the advantage of being individual. They are also less time-consuming and expensive than group auctions and can be conducted in the field at the point of sale^(22)^. Moreover, in contrast to theoretical mechanisms to elicit consumers’ WTP, BDM auctions use real products and a real exchange of money and do not suffer from hypothetical bias; therefore, they are more accurate, and the results display market behaviour better. Thus, the present study used the BDM auction method. The method was first described by Becker *et al.* ^(23)^ and can be combined with sensory evaluation^(17)^. Moreover, BDM auctions have already been successfully implemented in developing countries as the procedure is easy to understand by less educated respondents^(18,19)^.

**Fig. 1:**
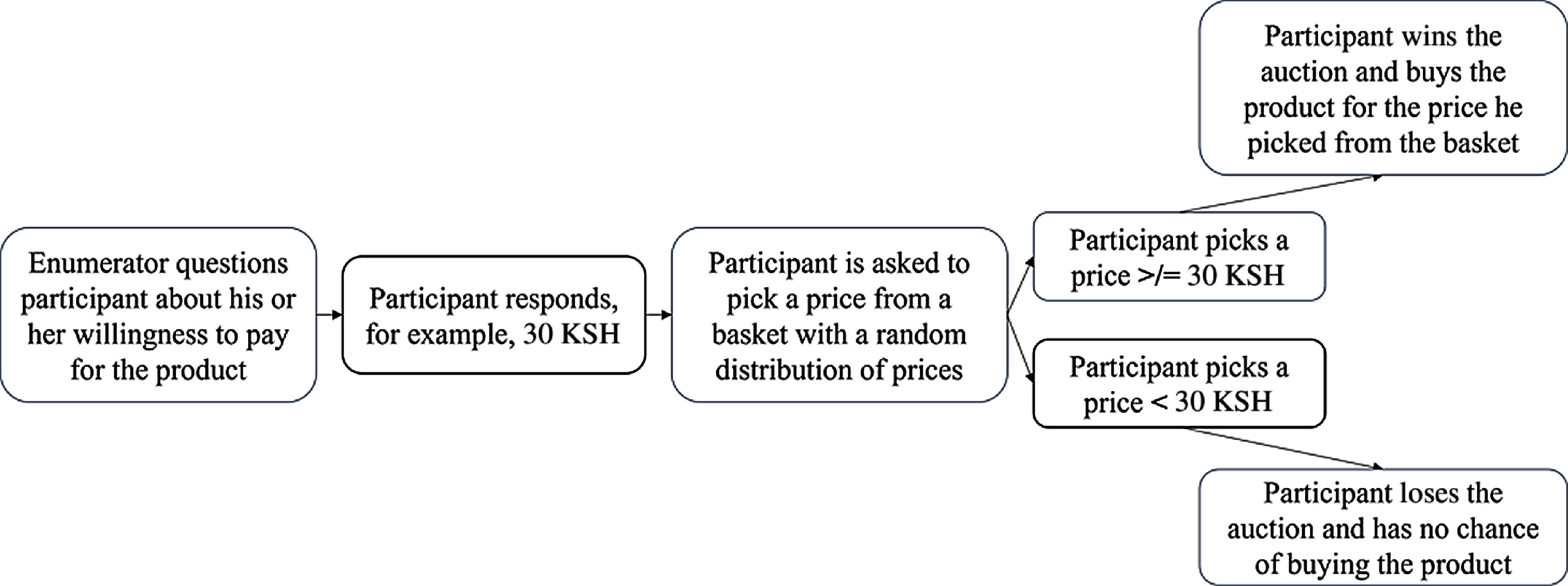
Brief scheme of the willingness to pay auction, own representation

The bid that a participant stated was compared with a randomly drawn price (Fig. 1). The prices used depended on the market price of similar products. The highest number in the lottery equalled twice the estimated market price. This led to the following distributions. In Kenya, the estimated market price was 30 KSH for each product; thus, the prices in the auction ranged from 5 to 60 KSH. In Tanzania, the expected market price for each product was 400 TSH; thus, the prices in the auction ranged from 50 to 800 TSH. In Uganda, the estimated market price for the jackfruit juice was 1100 UGX; thus, the numbers in the auction ranged from 200 to 2200 UGX.

The WTP auction consisted of the following. First, the enumerators explained the general procedure which entailed the respondents being asked to place a monetary value on the products they just tasted in detail. The respondents were encouraged to ask questions about the procedure. To ensure that respondents understood the procedure correctly, they were questioned about different scenarios and could proceed only after they had answered correctly. The respondents were then shown the fully packaged products they had just tasted and asked to state a bid for each of them (e.g. 50 g cowpea leaf soup mix and 250 ml guava nectar). Next, they were requested to draw a number from a basket of numbers. The numbers were generated around the expected WTP. The highest number was double the expected WTP. If the number drawn by the respondent was lower or as low as their originally stated WTP, they won the auction and had to purchase the product. Otherwise, they lost the auction and had no chance of buying the product. The respondents were asked first to make bids for each of the products before drawing numbers to ensure that the outcome of the first products would not influence the bidding on the second product.

### Empirical model

As participants could not state negative prices, we used a model that considered left-censored data. The Tobit model applied in the present survey was first described by Tobin^(24)^. It models the relationship between a censored continuous dependent variable y*_i_ and several independent variables x_i_, where y*_i_ is a latent variable observed for values greater than 0. 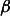 is a vector of estimable parameters. In the present survey, y*_i_ represented the WTP of participant i for one of the 6 products.
(1)

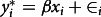




The dependent variable y_i_ was the WTP for each of the 6 fruit and vegetable products. The WTP was converted into dollars using the exchange rate at the time of the survey. To allow comparisons between the products, the purchasing power of each country was also considered, and the stated WTP was adapted accordingly. Therefore, the WTP in dollars was multiplied by the purchasing power parity factor of each country, respectively. The respective conversion factors were calculated by dividing the countries’ purchasing power parities by the countries’ gross domestic products. Data were obtained from the International Monetary Fund^(25)^. This led to a factor of 1·94 for the Kenyan products, 3·08 for the Tanzanian products and 3·42 for the Ugandan product. To account for differences that may have occurred due to the different natures of the markets sampled, the error term was clustered at the market level. The enumerator effect was included as a control variable.

In total, 8 variables were used to explain the WTP. The socio-demographic variables comprised age, sex, wealth, education, number of household members and location. Age was measured in years and grouped into quartiles (1 = 18 to 28; 2 = 29 to 38; 3 = 37 to 46; 4 = 46 and over). Wealth was an index that was calculated based on the WFP’s wealth index^(26)^ and considered participants’ ownership of livestock, land, access to water and sanitation facilities and types of floor and wall materials. Education represented the highest education level of the participant (1 = none; 2 = primary; 3 = secondary; 4 = tertiary). Location indicated whether a participant was questioned in a rural or an urban setting. In addition to the socio-demographic variables, sensory perception and an information treatment dummy were included in the model.

To measure the impact of sensory perception, the 5 food attributes that were analysed, namely, colour, aroma, texture in the mouth, taste and general appearance, were first condensed using principal component factor analysis with varimax rotation. For each product, one factor could be built out of the 5 characteristics (Table 2). The sampling adequacy was determined via Bartlett’s test and the Kaiser–Meyer–Olkin (KMO) criterion. The internal consistency was determined via Cronbach’s *α*. KMO values above 0·6 and Cronbach’s *α* values above 0·5 were considered acceptable.

**Table 2:** Results of the factor analysis on sensory perception

Item	Guava nectar	Cowpea leaf soup mix	Dried cashew apples	African nightshade relish	Dried African nightshade	Jackfruit juice
Colour	0·72	0·62	0·72	0·71	0·78	0·50
Aroma	0·68	0·77	0·76	0·77	0·78	0·64
Taste	0·75	0·86	0·71	0·81	0·82	0·61
Texture in the mouth	0·83	0·81	0·68	0·77	0·74	0·55
General appearance	0·80	0·84	0·72	0·81	0·84	0·71
Cronbach’s *α*	0·80	0·84	0·76	0·83	0·85	0·55
KMO	0·79	0·83	0·78	0·83	0·82	0·68

## Results

### Respondent characteristics

The respondent characteristics were similar across all 3 countries. Overall, slightly more women participated in the survey than men (Kenya: 59 %; Tanzania: 56 %; Uganda: 57 %). The respondents were young adults, with average ages of 36 years (Kenya), 39 years (Tanzania) and 37 years (Uganda), ranging from 18 to 88 years. All 3 countries have a young general population with average ages of 16 (Uganda), 17 (Tanzania) and 19 (Kenya)^(27)^. In each country, the average family size was approximately 4 people. The education rate was slightly higher in Kenya than in Tanzania and Uganda. In Kenya and Uganda, more than 50 % received at least a secondary education whereas in Tanzania, approximately 30 % of the participants received at least a secondary education. Approximately half of the participants in each country were questioned in rural areas (Kenya: 52 %, Tanzania: 53 % and Uganda: 46 %). A Kruskal–Wallis test was conducted to determine if participants’ socio-demographic characteristics differed significantly between the 3 countries. The test showed that there are differences between the countries in age, education and number of household members. The participants in Tanzania were significantly older than the participants in Kenya and Uganda (*P* = 0·001 and *P* = 0·002, respectively). The Kenyan participants were living in household with less members than the Tanzanian and Ugandan participants (*P* = 0·000 and *P* = 0·000, respectively). The Kenyan and Ugandan participants were better educated (*P* = 0·000 and *P* = 0·000, respectively) and wealthier (*P* = 0·000 and *P* = 0·000, respectively) than the Tanzanian participants. In all 3 countries, nearly 50 % of the participants received additional information about the products.

Moreover, we found socio-demographic differences between urban and rural participants. In Kenya and Uganda, the urban participants were significantly younger (*P* = 0·021 and *P* = 0·036, respectively) and were living with fewer household members (*P* = 0·055 and *P* = 0·006, respectively). The education rate was higher among the urban participants in Kenya and Tanzania (*P* = 0·000 and *P* = 0·017, respectively), and the urban participants were wealthier in Tanzania (*P* = 0·000) (see online Supplemental Table A.2).

Furthermore, we found sex differences. Significantly more men than women were questioned at an urban market in Uganda (*P* = 0·000). The female participants in Kenya and Uganda were significantly older than the male participants (*P* = 0·013 and *P* = 0·015, respectively), while the male participants in all 3 countries were better educated (*P*_Kenya_ = 0·000, *P*_Tanzania_ = 0·073 and *P*_Uganda_ = 0·044, respectively) (see online Supplemental Table A.3).

### Sensory analysis

The results of the sensory analysis show that all 6 products were predominantly perceived as positive in all 5 categories (see online Supplemental Table A.4). The products mostly received results of *like it* and *like it very much*. A Kruskal–Wallis test was conducted to determine the differences in liking between products. The test showed that there were significant differences in all categories, although not between all products in each category. The colour of the cowpea leaf soup mix was rated significantly lower than the colours of the other products, except for the African nightshade relish. Both, the cowpea leaf soup mix and the African nightshade relish, had a deep green colour. The yellowish colour of the jackfruit juice was liked better than the colour of any other product; the same result was found regarding the texture in the mouth and the taste of this product. Although sensory attributes were scored for most products and categories slightly higher among urban consumers, we find only a few statistically significant differences.

### Willingness to pay

**Fig. 2:**
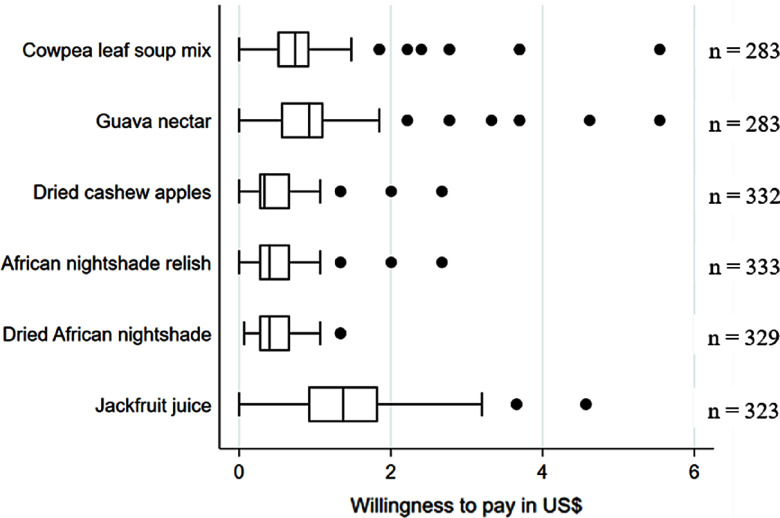
Mean willingness to pay for 6 fruit and vegetable products (values in US$, adapted by the purchasing power parity of the respective country in which the product was tested in)

Following the sensory analysis for all respective products, respondents were asked to state their WTP. The products were fully packaged and shown without labels to the participants. The results show a rather narrow distribution of the WTP for the cowpea leaf soup mix, dried cashew apples, African nightshade relish and dried African nightshade, indicating that participants had rather homogeneous opinions of the products (Fig. 2). The results for the jackfruit juice suggest quite different opinions about the products among the participants. For all products, it seems that participants have a more similar WTP among the lowest WTP quartile compared with the highest WTP quartile. The comparison between urban and rural consumers (Fig. A.6) shows a statistically significant higher WTP among urban consumers for all 6 products.

### Willingness to pay – Tobit model

To analyse the underlying factors influencing respondents’ WTP, a Tobit model was estimated for each of the products (Table 3). The results show that a positive sensory perception translates into a significantly higher WTP for all the products but the dried African nightshade. This might be because the dried African nightshade was prepared in a traditional way, and once prepared, it did not differ much from the African nightshade that was not dried before preparation and commonly consumed in the area. This leads to the conclusion that African nightshade is not predominantly consumed for its taste. For most of the products, the WTP decreased as age increased. A statistically significant effect was shown for jackfruit juice and guava nectar where an increase in the age group lowered the WTP by 0·11 US$ and 0·07 US$, respectively. We find that there is a tendency for wealthier and better-educated participants to be willing to pay more for the products. Additional information had a negative impact in all models except for guava nectar and cowpea leaf soup mix. The negative impact was statistically significant for the 2 African nightshade products. One reason could be that since these products are normally consumed in traditional settings, the information treatment may have triggered some lack of trust and, thus, resulted in a lower WTP. Being male increased the WTP in all models except for the model for dried African nightshade. The effect was found to be statistically significant for the dried cashew apples and the jackfruit juice, with marginal effects of 0·10 US$ for the dried cashew apples and 0·15 US$ for the jackfruit juice. For all products, the participants from urban areas were willing to pay more. The effect was especially strong for guava nectar. Being from an urban area increased the WTP by 0·47 US$. The number of people living in the household had a negative but not statistically significant effect for all products except the African nightshade relish. The interaction between wealth and sex decreased consumers’ WTP for all products but the African nightshade relish. The effect was statistically significant for the jackfruit juice.

**Table 3:** Tobit analysis of respondents’ willingness to pay for fruit and vegetable products

	Kenya				Tanzania						Uganda	
	Cowpea leaf soup		Guava nectar		Dried cashew apples		African nightshade relish		Dried African nightshade		Jackfruit juice	
	Coefficient	SD	Coefficient	SD	Coefficient	SD	Coefficient	SD	Coefficient	SD	Coefficient	SD
Sensory analysis (factor)	0·173**	0·076	0·115*	0·068	0·044***	0·016	0·027*	0·015	0·011	0·023	0·073***	0·026
	0·024		0·090		0·005		0·084		0·632		0·005	
Age groups	−0·008	0·031	−0·072*	0·043	0·013	0·018	0·004	0·008	−0·007	014	−0·107***	0·019
	0·789		0·096		0·479		0·650		0·581		0·000	
Wealth (index)	0·224	0·152	0·151	0·195	0·109*	0·07	−0·011	0·077	0·047	0·054	0·432***	0·061
	0·142		0·440		0·098		0·888		0·386		0·000	
Received information	0·004	0·115	0·149	0·143	−0·013	0·023	−0·076***	0·029	−0·047*	0·028	−0·033	0·041
	0·973		0·299		0·567		0·010		0·089		0·412	
Education level	0·061	0·038	0·095*	0·055	0·006	0·044	0·032	0·033	0·003	0·022	0·027	0·03
	0·110		0·084		0·883		0·332		0·897		0·371	
Sex (female)	−0·182	0·113	−0·112	0·099	−0·101*	0·054	−0·02	0·033	0·008	0·037	−0·146***	0·022
	0·108		0·257		0·062		0·543		0·826		0·000	
Location (urban)	0·352*	0·195	0·469***	0·136	0·028	0·058	0·04	0·049	0·064	0·069	0·003	0·068
	0·072		0·001		0·632		0·417		0·349		0·961	
No. of household members	−0·035	0·027	−0·029	0·019	−0·009	0·006	0·002	0·007	−0·001	0·005	0·001	0·01
	0·195		0·135		0·173		0·754		0·805		0·937	
Sex (female) #Wealth (index)	−0·125	0·077	−0·105	0·102	−0·055	0·038	0·014	0·037	−0·019	0·045	−0·202***	0·032
	0·107		0·305		0·144		0·709		0·595		0·000	
*n*	283		283		332		333		329		323	
Mean	0·94	1·08	0·98	0·83	0·49	0·42	0·46	0·46	0·54	0·41	1·51	1·16
pseudo *R* ^2^	0·139		0·089		0·133		0·296		0·164		0·079	

*P* – values in parentheses.

* *P* < 0 1.

** *P* < 0 05.

*** *P* < 0 01.

## Discussion

The main objective of this study is to assess consumers’ demands for fruit and vegetable products in East Africa. Previous studies on this topic have demonstrated the power of combining sensory analysis and BDM to address the objective^(17,19)^. The most important finding of the present survey is the positive perception of different highly nutritious fruit and vegetable products across Kenya, Tanzania and Uganda. Considering the nutritional benefits of these products, this offers a great opportunity to increase their consumption. The analysis of the factors explaining the consumers’ WTP revealed several consistent findings among all 6 products. These are important results for further marketing and dissemination of the products. As expected, *sensory liking* had a high influence on consumers’ WTP. This finding has to be understood in the context of the mean sensory rating between *like it* and *like it very much*. Moreover, our results confirm the influence of socio-economic factors on the WTP for fruit and vegetable products, which is consistent with other studies conducted in sub-Saharan Africa but to different extents^(10,28)^.

While Okello *et al.* ^(10)^ showed a higher WTP for value-added cowpea leaves among elderly people and women, the present survey found that *age* and being *female* negatively influenced the WTP for most of the surveyed products. This might be because the products being analysed by Okello *et al*.^(10)^ were simply sun-dried and, thus, resembled the fresh produce more than the products in the present survey. This also goes in line with our finding that being female did not decrease the WTP for the dried African nightshade, which resembles the product analysed by Okello *et al.* ^(10)^. Hence, we can conclude that women prefer processed products less than men, although this is not necessarily a causal relationship. Additionally, Van der Lans *et al*.^(9)^ found that women, in general, consume more vegetables than men and might, therefore, have a lower need to enhance their consumption patterns by adding processed fruits and vegetables. We speculate that women place higher value on the raw material and prefer home preparation or processing of their food. Women are presumed to have higher culinary skills and thus, to be less dependent on ready-to-eat products. These are important findings as women are most vulnerable to micronutrient deficiencies^(3)^.

Unlike previous studies that showed that additional *information* can increase consumers’ WTP for food products^(21,29)^, we found the information to barely have an effect. The present survey presented information as a combination of images and texts while previous studies used radio messaging or simple text and only focussed on nutritional information. This finding highlights the importance of determining how nutrition information needs to be placed to appeal to consumers and concludes that the effect to be expected from before purchase information is limited at best. This issue that was already highlighted by Lagerkvist *et al.* ^(30)^, who found that detailed information about nutritional benefits decreased consumer acceptance of biofortified orange-fleshed potato in Kenya.

According to the nutritional transition, food consumption in East Africa (and globally) has shifted towards more processed foods. This includes rising demand for food groups such as soups, nectars or fruit snacks, and products that were analysed in the present survey^(13–15)^. A major challenge of the current trend is the low nutritional value but high sugar and fat concentrations of many processed products and the consequential health issues^(13)^. The products in the present survey fit into the current demand but also provide great nutritional value, as they are rich in micronutrients such as vitamin A, Fe and/or Zn. Undersupply of these nutrients can be observed in large parts of East Africa, and their sufficient intake is highly dependent on adequate dietary diversity^(31)^.

Consumers from *urban* areas, who are expected to have better access to and knowledge of already existing processed foods, were willing to pay more for the products. This finding is in contrast to findings for fresh vegetables^(32)^, which show low acceptance of African leafy vegetables among urban dwellers. These findings also showed that taste hinders demand. Based on our findings, it seems that processing can overcome this obstacle. This finding is in line with previous studies demonstrating the potential of processing to enhance the acceptability of cashew apples^(12)^. Knowledge concerning the preparation of fresh African leafy vegetables will likely be higher in rural areas; and thus, the need for processed products will be lower. However, as seasonality causes shortages in fresh produce and increases malnutrition^(4)^, familiarity with processed products should increase in rural areas. As most of the products do not require difficult processing, it is also possible to educate rural consumers on the processing of healthy food items at the household level.

Additionally, *wealth* had a positive impact on the WTP, which highlights the importance of setting the price for the products carefully, to avoid excluding poorer households. Finding profitable markets for new processors and simultaneously reaching the very poor with improved nutrition is a difficulty that was already emphasised by De Groote *et al.* ^(17)^. They suggest that profitable enterprises should be established first among wealthier consumers and that successful operations should allow them to reach poorer households. The technologies used to produce the products of the present study, however, were especially chosen because they can be easily implemented in rural settings, to improve nutrition among the most vulnerable population groups. Additionally, the raw material used for production can be sourced directly in the rural areas, and establishing enterprises in those regions will lower transportation costs, which will make the products affordable for the poor. It needs to be noted that according to the results of the interaction between wealth and sex, we find that the positive wealth effect is more pronounced for men.

The *number of family members* living in the household had a negative effect on the WTP. The products were packaged in small quantities and, thus, were unlikely to be sufficient for larger families. The packaging size had convenience purposes for the sake of this survey. Future selling in the market should provide different packaging sizes to appeal to different numbers of household members. Additionally, food preferences will vary more in larger families, thus making it more difficult to find a product that appeals to everyone.

The WTP also increased with *education*. Better-educated people are assumed to already have better knowledge of dietary health implications and are, thus, more interested in choosing healthy food.

Several limitations of the study need to be discussed. First, the results show tendencies for certain consumer groups but no constant statistically significant results. Further studies are necessary to confirm our findings. Second, the survey did not include children. As micronutrient deficiencies are also widespread among this population group^(3)^, future research could, for example, address possibilities to include the products in children’s school food. Further subgroups such as pregnant women or women in childbearing age could also be of interest in future studies. Third, the study did not cover differences in the WTP between seasons. Assessing differences in perception between the lean and peak seasons may be an interesting topic in future research. Fourth, it might be possible that despite their limitations, the information treatments could be effective among subgroups, who are more pronounced to media channels or radio. This was, however, not analysed in the present study and should be considered in future research. Finally, it is important to note that while the products presented here are rich in nutrients, they play only one part to improve health and cannot erase malnutrition alone. They must be included smartly in the daily and healthy diets. Sarfo *et al.* (unpublished results) tested minimally processed fruits and vegetables, including cowpea leaf soup mix, in a modelled diet for women and children in rural Tanzania. In their results, the minimally processed fruits and vegetables substantially reduced the diet cost of women and children between 12 and 23 months. Additionally, nutrient gaps such as those for Fe, Zn, vitamin A, vitamin C and vitamin B_2_ were bridged with the addition of the minimally processed fruits and vegetables to the diets.

Moreover, we learned some lessons from the set-up of the survey. While approaching people in open markets, we were able to question many participants, but the set-up was highly subject to environmental effects. Sudden weather changes forced interruptions in questioning and aggravated the separation of waiting people from participants. To ensure independent results, many participants had to be dropped from the survey. In addition, participants came to the market to do their grocery shopping and were often in a hurry to finish the survey. It is, thus, recommended to preselect and invite consumers to less crowded and better controllable environments. This would also allow for a more sensitive selection of the participants to include representative numbers from different socio-demographic groups. The present survey is representative of the selected markets but not of the selected countries, so some caution about the external validity of the results should be considered. The participant’ characteristics are summarised in Table 2.

## Conclusion and policy implications

The sensory analysis indicates high appreciation for all 6 fruit and vegetable products and translates into higher WTP. We concluded that there is demand and a market for processed fruit and vegetable products based on indigenous raw materials in East Africa. The products, thus, have promising potential to improve nutrition, especially during off-season conditions when access to fresh produce is limited. Our analysis shows that similar socio-demographic characteristics influence the demand for the diverse spectrum of products analysed in this study. While fresh African leafy vegetables are mostly consumed among elderly, poorer and rural populations^(33)^, processing enhances acceptance among younger, wealthier and urban dwellers. This offers wider sales opportunities but also calls for careful marketing strategies to ensure inclusion of the most severely impacted populations. Surprisingly, image-based information on nutritional and/or shelf life benefits was not helpful to create additional demand. Future surveys should assess how to promote the products best. Additionally, the images must be adapted to anticipate current drawbacks.

To conclude, it is encouraging that the products were so well received, and many participants were willing to pay a reasonable price. To our knowledge, this is the first study that comprehensively analysed consumer demand for 6 different products across 3 different countries. Our findings that women and rural participants, who are often most affected by malnutrition, are more reserved towards the products implies that sole processing is not the solution; we need to improve the perception among these population groups. We also find that younger, male and urban consumers are more willing to buy the products. These groups are likely to have less knowledge of preparation and cooking techniques^(32)^ and could, thus, greatly profit from processed fruit and vegetable products.

As we have shown consumers interest in nutritious fruit and vegetable products obtained from local plants, supporting this business should be interesting for policymakers. The products are suitable for diverse groups of the population, ranging from children to the elderly. However, our research shows that interventions are necessary to reach all consumer segments, especially those impacted most severely by micronutrient deficiencies. Women and the rural population are less interested in the novel fruit and vegetable products independent of wealth, although they are more often deficient in some of the micronutrients the products offer. Community events can be an opportunity to advertise the products, how they are created, and raise familiarity among the rural population. Additionally, educating women on processing techniques could improve dissemination and acceptance among this consumer group.

Considering that sensory characteristics play a significant role in shaping consumer demand, supporting research on product development might enhance the utilisation of these fruits and vegetables. It is important to note that all government support of fruit and vegetable processing should be linked to retaining health aspects in the final products.

## Supplementary material

For supplementary material accompanying this paper visit https://doi.org/10.1017/S136898002100478X. click here to view supplementary material
