# Supplementary material for: Consumer demand for novel fruit and vegetable products with extended shelf lives in East Africa: a multinational multi-product analysis
Source: Public Health Nutr. 2021 Dec 10;25(6):1472–82. doi: 10.1017/S136898002100478X (PMC9991703; doi:10.1017/S136898002100478X)
Supplement: Supplementary file 1 [file S136898002100478Xsup001.pdf]

Appendix

Consumer demand for novel fruit and vegetable products with extended shelf lives in East Africa: A multinational multiproduct analysis

Table A.1: Participant characteristics, by information

Variable	With information mean (Std.)	Without information mean (Std.)	Difference (p-value)
Sex (Female)	58 %	57 %	0.772
Age (years)	38.41 (12.47)	37.72 (12.95)	0.285
No. of household members	4.27 (2.16)	4.17 (2.19)	0.421
Education level (1 = none; 2 = primary; 3 = secondary; 4 = tertiary)	2.48 (0.72)	2.52 (0.78)	0.469
Wealth (Index)	0.002 (1)	-0.002 (1)	0.332
Location (urban)	50 %	48 %	0.551
Sensory perception			
Cowpea leaf soup mix	0.12 (0.91)	-0.13 (1.07)	0.037**
Guava nectar	-0.03 (0.99)	0.03 (1.01)	0.814
Dried cashew apples	-0.04 (0.97)	0.04 (1.03)	0.375
African nightshade relish	-0.03 (1.06)	0.03 (0.93)	0.865
Dried African nightshade	-0.05 (0.96)	0.05 (1.04)	0.334
Jackfruit juice	-0.11 (1.04)	0.11 (0.94)	0.053*
N	482	460	

Note: * $p < 0.1$ and ** $p < 0.05$ according to Mann-Whitney-U-Test

Figure A.1: Cowpea leaf soup (Kenya)

Insufficiency in micronutrients such as vitamin A, vitamin C or iron has been reported in Kenya. With insufficient intake of micronutrients, we are more prone to night blindness, tiredness, and infection. Eating a cowpea leaf soup mix can help to improve your health, because it is a good source of vitamin A, iron, and zinc. Cowpea leaves are even more nutritious than kales which are commonly consumed. While fresh cowpea leaves are only available during harvest season, the cowpea leaf soup mix has a long shelf-life and is available all year-round, independent of the cowpea growing season. This allows you to consume it even when fresh cowpeas are not available. The cowpea leaf soup mix can be stored over several month and will remain safe. Preparing a cowpea soup mix is easy and fast. Once the water boils, the soup is ready within five minutes. No special cooking skills are needed. This saves time.

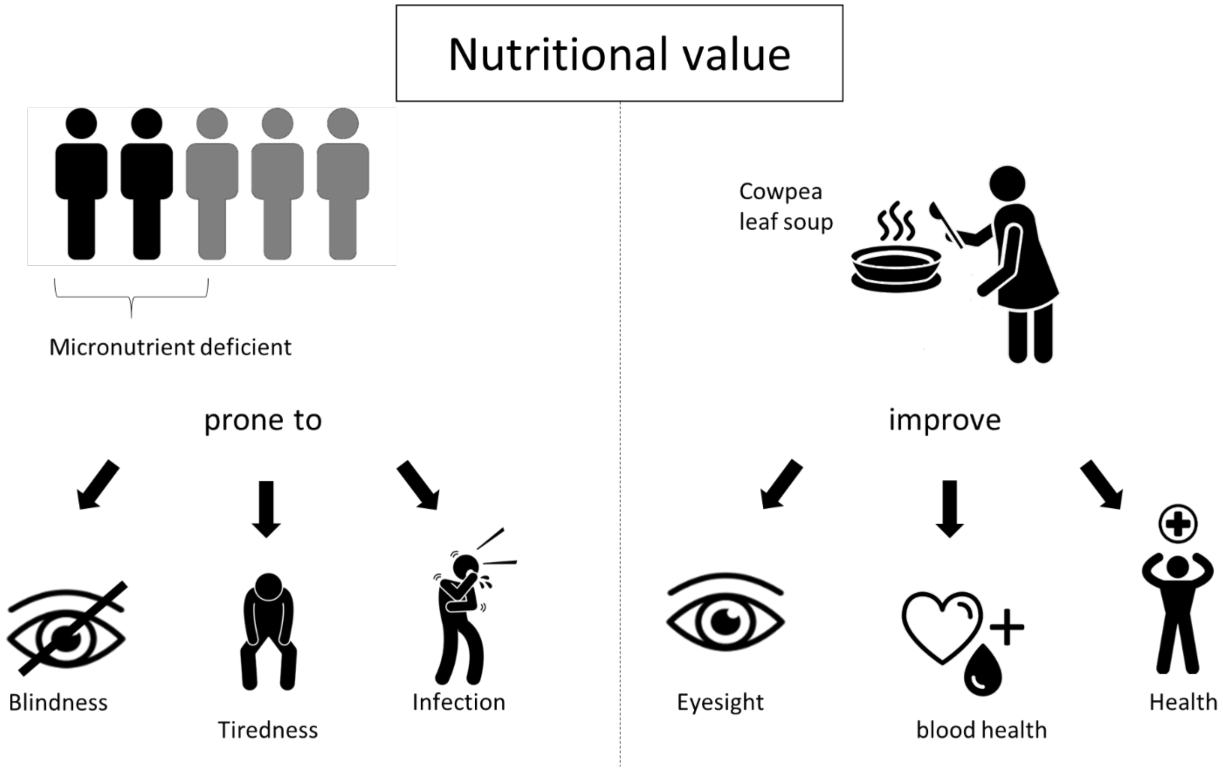

Shelf-life

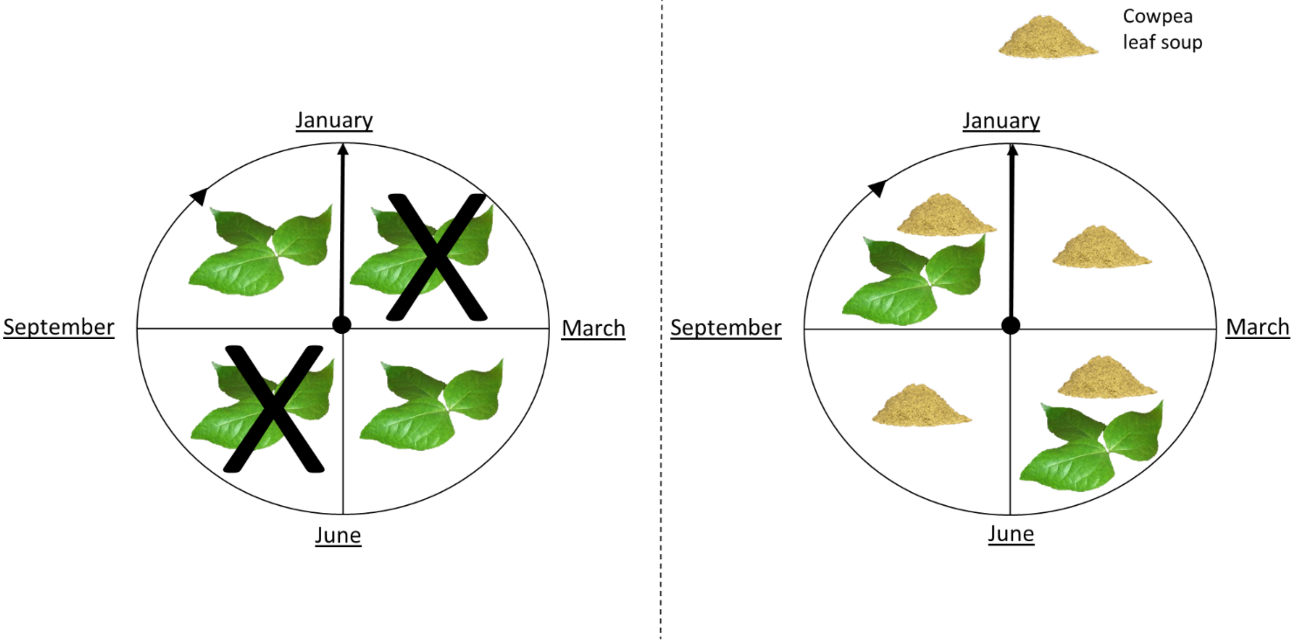

Convenience

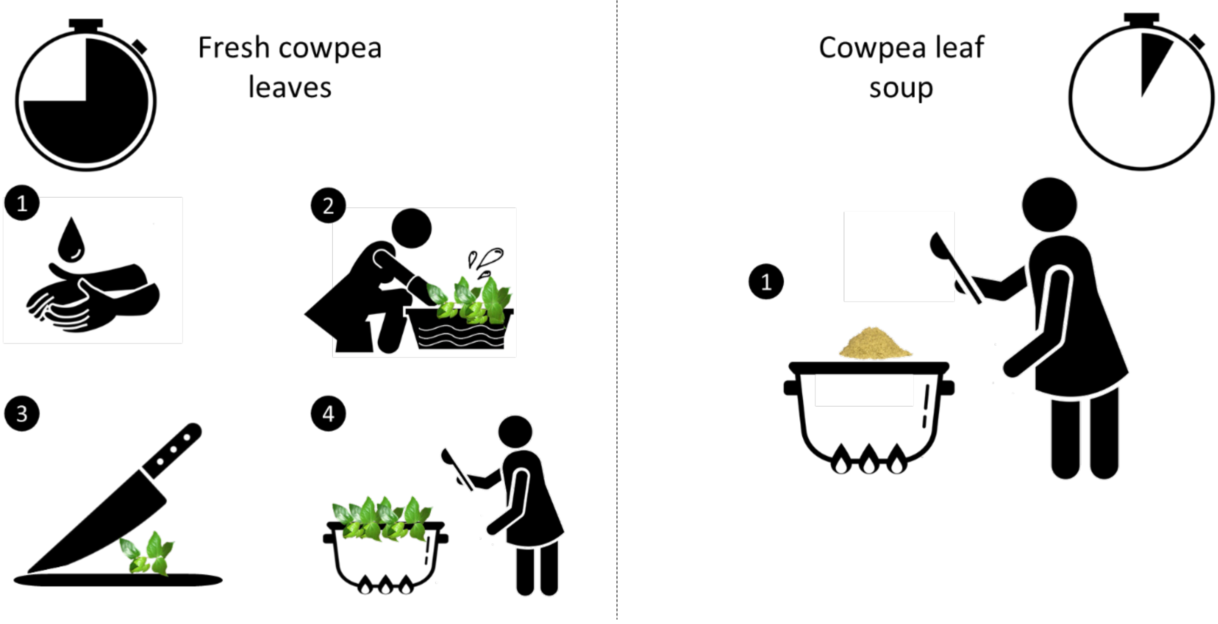

Table A.2: Descriptive statistics of participants by country and location

	Kenya			Tanzania			Uganda		
	**Rural**	**Urban**		**Rural**	**Urban**		**Rural**	**Urban**	
Variable	Mean (Std.)	Mean (Std.)	Difference (p-value)	Mean (Std.)	Mean (Std.)	Difference (p-value)	Mean (Std.)	Mean (Std.)	Difference (p-value)
Female	62 %	55 %	0.195	54 %	59 %	0.397	67 %	48 %	0.000***
Age (years)	38.95 (13.7)	34.97 (11.37)	0.021**	40.53 (13.03)	39.22 (14.4)	0.458	38.83 (13.39)	35.45 (11.30)	0.036**
No. of household members	3.97 (2.04)	3.51 (1.84)	0.055*	4.33 (2.04)	4.4 (1.67)	0.545	4.96 (2.82)	4.09 (2.21)	0.006***
Education level (1 = none; 2 = primary; 3 = secondary; 4 = tertiary)	2.60 (0.75)	2.99 (0.82)	0.000***	2.16 (0.62)	2.33 (0.63)	0.017**	2.47 (0.72)	2.54 (0.74)	0.316
Wealth (Index)	-0.25 (1.19)	0.34 (0.51)	0.328	-0.54 (1.31)	0.32 (0.56)	0.000***	-0.03 (1.03)	0.25 (0.69)	0.764
Information (yes)	49 %	52 %	0.631	51 %	53 %	0.666	51 %	51 %	0.889
Sensory perception									
Cowpea leaf soup mix	-0.03 (1.06)	0.03 (0.94)	0.885						
Guava nectar	-0.1 (1.06)	0.11 (0.92)	0.088*						
Dried cashew apples				-0.07 (0.98)	0.08 (1.02)	0.051*			
African nightshade relish				0.03 (0.95)	-0.04 (1.06)	0.662			
Dried African nightshade				-0.07 (1.04)	0.08 (0.95)	0.048**			

Jackfruit juice							0.04<br>(1.04)	-0.04<br>(0.95)	0.132
N	149	134		177	156		150	173	

Note: * $p < 0.1$, ** $p < 0.05$, and *** $p < 0.01$ according to Mann-Whitney-U-Test

Table A.3: Descriptive statistics of participants by country and sex

	Kenya			Tanzania			Uganda		
	**Men**	**Women**		**Men**	**Women**		**Men**	**Women**	
Variable	Mean (Std.)	Mean (Std.)	Difference (p-value)	Mean (Std.)	Mean (Std.)	Difference (p-value)	Mean (Std.)	Mean (Std.)	Difference (p-value)
Location (urban)	52 %	44 %	0.195	44 %	48 %	0.379	64 %	45 %	0.000***
Age (years)	35.02 (12.54)	38.49 (12.78)	0.013**	40.51 (13.59)	39.47 (12.06)	0.653	35.69 (13.38)	38.04 (11.55)	0.015**
No. of household members	3.41 (1.85)	3.99 (2.01)	0.014**	4.42 (1.98)	4.31 (1.80)	0.490	4.41 (2.74)	4.55 (2.39)	0.183
Education level (1 = none; 2 = primary; 3 = secondary; 4 = tertiary)	3.04 (0.78)	2.60 (0.78)	0.000**	2.32 (0.61)	2.18 (0.64)	0.073*	2.60 (0.71)	2.44 (0.74)	0.044**
Wealth (Index)	0.07 (0.94)	0.00 (1)	0.82	-0.06 (1.06)	-0.21 (1.16)	0.065*	0.17 (0.82)	0.08 (0.92)	0.105
Information (yes)	46 %	53 %	0.237	54 %	50 %	0.486	51 %	51 %	0.908
Sensory perception									
Cowpea leaf soup mix	-0.05 (1.09)-	0.03 (0.93)	0.772						
Guava nectar	0.04 (0.93)	0.03 (1.05)	0.233						
Dried cashew apples				-0.17 (1.15)	0.13 (0.85)	0.035*			
African nightshade relish				-0.05 (1.05)	0.04 (0.96)	0.502			
Dried African nightshade				-0.00 (0.99)	0.00 (1.01)	0.753			

Jackfruit juice							-0.16<br>(1.04)	0.12<br>(0.95)	0.005***
Willingness to pay (US $)									
Cowpea leaf soup mix	1.04<br>(0.91)	0.79<br>(0.59)	0.083*						
Guava nectar	1.11<br>(0.91)	0.88<br>(0.66)	0.047**						
Dried cashew ap-ples				0.51<br>(0.42)	0.42<br>(0.25)	0.332			
African nightshade relish				0.44<br>(0.33)	0.41<br>(0.22)	0.964			
Dried African nightshade				0.50<br>(0.29)	0.51<br>(0.30)	0.706			
Jackfruit juice							1.59<br>(0.72)	1.39<br>(0.07)	0.002***
N	116	167		146	189		140	183	

Note: * $p < 0.1$, ** $p < 0.05$, and *** $p < 0.01$ according to Mann-Whitney-U-Test

Table A.4: Result of the sensory analysis

	Cowpea leaf soup mix	Guava nectar	Dried cashew apples	African night-shade relish	Dried African nightshade	Jackfruit juice
Color	3.95 ± 1.02^{a}	4.18 ± 0.93^{b}	4.32 ± 0.63^{bc}	4.10 ± 0.66^{ad}	4.33 ± 0.68^{bce}	4.56 ± 0.68^{f}
Aroma	4.15 ± 0.97^{a}	4.35 ± 0.91^{b}	4.29 ± 0.65^{ac}	4.04 ± 0.75^{d}	4.32 ± 0.66^{ace}	4.49 ± 0.76^{bf}
Texture in the mouth	4.15 ± 0.98^{a}	4.41 ± 0.78^{b}	4.32 ± 0.69^{ac}	4.16 ± 0.72^{ad}	4.34 ± 0.68^{ce}	4.53 ± 0.77^{f}
Taste	4.23 ± 1.07^{a}	4.62 ± 0.68^{b}	4.61 ± 0.57^{b}	4.38 ± 0.72^{ad}	4.56 ± 0.69^{bce}	4.72 ± 0.62^{f}
General appearance	4.33 ± 0.86^{a}	4.56 ± 0.71^{b}	4.69 ± 0.54^{c}	4.41 ± 0.70^{ad}	4.63 ± 0.64^{bce}	4.72 ± 0.51^{cef}
N	283	283	332	333	329	323

Note: different letters a, b, c, d, e, f reflects a significant difference in a characteristic between products ($p < 0.05$) according to Kruskal -Wallis and Dunnet-T; scales for all five categories ranged from 1 = dislike it very much to 5 = like it very much

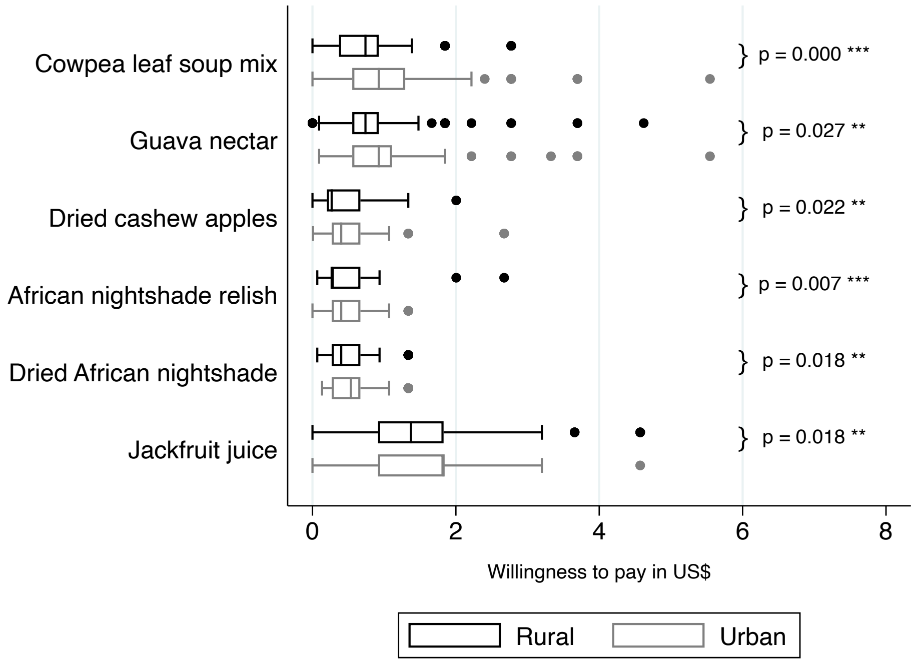

Figure A.6: Mean willingness to pay for six fruit and vegetable products, by region (values in US$, adapted by the purchasing power parity of the respective country the product was tested in); ** $p < 0.05$, and *** $p < 0.01$ according to Mann-Whitney-U-Test between urban and rural consumers
